# Live Visualization of Hemagglutinin Dynamics during Infection by Using a Novel Reporter Influenza A Virus

**DOI:** 10.3390/v12060687

**Published:** 2020-06-26

**Authors:** Luiz Gustavo dos Anjos Borges, Giuseppe Pisanelli, Oyahida Khatun, Adolfo García-Sastre, Shashank Tripathi

**Affiliations:** 1 Department of Microbiology, Icahn School of Medicine at Mount Sinai, New York, NY 10029, USA; luizgaborges@gmail.com (L.G.d.A.B.); giuseppe.pisanelli@mssm.edu (G.P.); Adolfo.Garcia-Sastre@mssm.edu (A.G.-S.); 2 Global Health and Emerging Pathogens Institute, Icahn School of Medicine at Mount Sinai, New York, NY 10029, USA; 3 Department of Veterinary Medicine and Animal Production, University of Naples Federico II, Via Federico Delpino 1, 80137 Naples, Italy; 4 Department of Microbiology & Cell Biology, Indian Institute of Science, Bengaluru 560012, India; oyahidak@iisc.ac.in; 5 Centre for Infectious Disease Research, Indian Institute of Science, Bengaluru 560012, India; 6 Department of Medicine, Division of Infectious Diseases, Icahn School of Medicine at Mount Sinai, New York, NY 10029, USA; 7 The Tisch Cancer Institute, Icahn School of Medicine at Mount Sinai, New York, NY 10029, USA

**Keywords:** influenza, hemagglutinin, tetra cysteine tag, biarsenical labeling, live imaging

## Abstract

Live visualization of influenza A virus (IAV) structural proteins during viral infection in cells is highly sought objective to study different aspects of the viral replication cycle. To achieve this, we engineered an IAV to express a Tetra Cysteine tag (TC tag) from hemagglutinin (HA), which allows intracellular labeling of the engineered HA protein with biarsenic dyes and subsequent fluorescence detection. Using such constructs, we rescued a recombinant IAV with TC tag inserted in HA, in A/Puerto Rico/8/1934(H1N1) background (HA-TC). This recombinant HA-TC tag reporter IAV was replication-competent; however, as compared to wild type PR8 IAV, it was attenuated in multicycle replication. We confirmed expression of TC tag and biarsenical labeling of HA by immunofluorescence assay in cells infected with an HA-TC tag reporter IAV. Further, we used this reporter virus to visualize HA expression and translocation in IAV infected cells by live confocal imaging. We also tested the utility of the HA-TC IAV in testing chemical inhibitors of the HA translocation. Overall, HA-TC IAV is a versatile tool that will be useful for studying viral life cycle events, virus-host interactions, and anti-viral testing.

## 1. Introduction

Influenza A viruses are enveloped, negative-sense segmented RNA viruses of the family *Orthomyxoviridae.* The viral genome encodes more than a dozen proteins, of which Hemagglutinin is the major structural protein that binds to the sialic acid moieties on the host cell surface to facilitate viral entry. Influenza viruses are major human pathogens that cause respiratory infection and disease, commonly called Flu. Among all human viral pathogens, influenza is one of the most extensively studied, especially aspects of the virion and viral protein structures, viral replication cycle, and mechanisms of viral pathogenesis. Hemagglutinin (HA), the major surface glycoprotein, is a key immunogen of natural immunity and vaccine approaches and plays an important role in virus entry and for determining host tropism [1]. HA is a type I transmembrane protein that is synthesized as precursor HA₀ (75 kDa) in the endoplasmic reticulum (ER) and undergoes N-glycosylation and palmitoylation. Subsequently, HA₀ is transported to the plasma membrane through the ER-Golgi network [2]. During this process or upon release from the cells HA₀ is cleaved by intracellular or extracellular proteases into HA₁ (55 kDa) and HA₂ (25 kDa) subunits. HA₁ has the receptor binding site, which helps in entry by binding to the terminal α-2,3- or α-2,6-linked sialic acid moieties present on the host cell surface. HA₂ is the membrane-anchored fraction and helps in membrane fusion with its fusion peptide [1,2]. Among influenza A virus (IAV) structural proteins, extensive research has been done on IAV HA structure and conformational changes during viral entry and HA post-translational modification. Comparatively, there is a dearth of literature on the aspects of HA trafficking through the ER-Golgi network to the virus budding sites on the cell membrane. Live imaging of HA in IAV infected cells can allow a detailed study of this aspect.

In this study, we explored the possibility of live imaging of HA during IAV infection in cells. This process would require enabling HA fluorescence through the expression of a reporter tag. HA is the most variable proteins among IAV proteins, and it has been shown that it allows the insertion of foreign gene sequences and expression through an engineered virus [3,4,5,6]. Although in-frame fusion of any fluorescent protein (e.g., GFP) helps in visualization, the large size of GFP (~25 kDa) may affect normal trafficking. To minimize size issues, we applied a small tag labeling system developed by Roger Tsien and Colleagues [7]. This tag, called Tetra cysteine tag, has two pairs of cysteines separated by two amino acids (-CCXXCC-), which can be fused in-frame to the protein of interest. The tetra cysteine tag gives fluorescence upon binding with membrane-permeable fluorescein derivatives, Fluorescein Arsenical Hairpin Binder (FIAsh), or resorufin arsenical hairpin binder (ReAsh). Neither the tag nor these derivatives are fluorescent; however, binding of these compounds with the tag gives fluorescence with reduced background. The small size of the tag does not affect the functionality of the protein; also 1:1 stoichiometry of tag: FIAsh has reduced background. This tag has been used in live imaging of structural proteins HIV, VSV, Ebola, Blue tong virus, and even for Influenza NS1 and NP proteins [8,9,10,11,12,13,14,15]. Here, we report successful expression of TC tag inserted in-frame in IAV HA protein in cells infected with the engineered reporter IAV. We went on further to conduct 4-dimensional tracking of HA during IAV infection cycle. We were able to get information on spatial and temporal distribution of HA during infection progression. We also demonstrated the utility of the reporter IAV in studying the effect of chemical inhibitors on different measurements of HA accumulation and trafficking.

## 2. Materials and Methods

### 2.1. Cell Lines, Antibodies, and Drugs

Human embryonic kidney (293T) cells were maintained in DMEM supplemented with 10% FBS and 1000 units/mL penicillin/streptomycin. Madin–Darby canine kidney (MDCK) cells were maintained in MEM supplemented with 10% FBS and penicillin/streptomycin. Reagents for cell culture were purchased from Gibco Life Technologies. Anti-IAV HA and Anti-IAV NP mouse monoclonal antibodies were obtained from Hybridoma core of Mount Sinai School of Medicine. HRP conjugated anti-beta actin antibody was procured from Abcam (AC-15; #ab49900; Cambridge, USA). Brefeldin A (#B7651), Golgicide (#G0923) and Tunicamycin (#654380) were procured from Sigma-Aldrich (St. Louis, USA). Brefeldin A was used at working concentration of 100 nM. Golgicide was used at working concentration of 10 µM. Tunicamycin was used from 0.2 to 2 µM working concentration in different experiments. The drug doses were chosen based on literature and published cytotoxicity data [16,17,18,19].

### 2.2. IAV Infection of Cells

MDCK cells were infected with virus inoculum in DMEM containing 0.2% bovine serum albumin (BSA, Thermo fisher, Waltham, USA). After 1 h, the infection medium was removed and the cells were incubated with 2 mL of DMEM–0.2% BSA and supplemented with 1 µg/mL of TPCK (tosyl sulfonyl phenylalanyl chloromethyl ketone)-treated trypsin/mL to allow the production of fusion-competent viruses. Supernatants were collected at indicated time points after infection, and viral titers were determined by plaque assay in MDCK cells.

### 2.3. Plaque Assay

MDCK cells were seeded in six-well plates, 1 day before infection, at a dilution of 10^6^ cells/well. The next day, the cells were washed once with 2 mL PBS and incubated with virus diluted in 200 μL PBS containing 0.3% bovine albumin (BA) and 1000 u/mL penicillin/streptomycin (PBS/BA; MP Biochemicals) for 1 h at 37 °C with frequent shaking. After incubation, the virus inoculum was removed and overlaid with MEM containing a 0.6% oxoid agar and 1 μL/mL L-tosylamido-2-phenyl ethyl chloromethyl ketone (TPCK-treated trypsin (Sigma, St. Louis, USA). The plaques were visualized by staining with Crystal Violet. The limit of detection of plaque assay was 10^10^ pfu/mL.

### 2.4. Virus Rescue

Virus rescues were performed as previously described by the eight-plasmid reverse genetics system in PR8 IAV background [20]. HA-TC pDZ plasmid along with 7 pDZ plasmids expressing other viral proteins was co-transfected in co-culture of HEK293T and MDCK cells. Culture supernatant was used 48 h later to plaque purify the virus from MDCK cells and passaged in 11 day old embryonated chicken eggs to generate a working stock. Rescued recombinant HA-TC PR8 IAV was confirmed by Sanger sequencing of the HA segment, and viral titers were determined by plaque assay in MDCK cells.

### 2.5. Virus Like Particle Formation and Hemagglutination Assay

For VLP formation HEK 293T cells were plated in 10 cm dish to achieve 50% confluency on the next day, when they were transfected with pDZ-HA-TC), pDZ-NA and pDZ-M plasmids in 2:2:1 ratio (total 5 µg DNA). Supernatant was collected 48 h later, clarified by centrifugation and filtration through 0.45µm filter. The culture supernatant was layered on 20% sucrose gradient in NTE buffer and concentrated by ultracentrifugation (112,600× *g*, 2 hrs, 4 °C, in a SW28 rotor (Beckman Coulter, Fullerton, California). The VLP pellet was resuspended in NTE buffer (100 mM NaCl, 10 mM Tris-Cl (pH 7.4), 1 mM EDTA). For hemagglutination assay, VLP solution was incubated with TPCK-treated trypsin (Sigma) at 1 µg/mL working concentration at 37 °C for 1 h. Subsequently 100 µL VLP solution was added to first well and 2-fold serial dilution was made with PBS in next series of wells in a V bottom 96 well plate (Nunc). To this 50 µL Sheep RBC (1% solution in PBS) was mixed and incubated at room temperature for 1 h.

### 2.6. Immunostaining

MDCK cells were infected with IAV at indicated multiplicity of infection (MOI). At indicated time point, the cells were washed with PBS and fixed in 1 mL 4% formaldehyde (methanol-free) for 10 min. After permeabilization with 0.5% Triton X-100 in PBS, the cells were stained in PBS containing 2% BSA with an anti-NP antibody. The cells were washed twice in PBS and stained with secondary antibody conjugated to Alexa-588 (Invitrogen, Waltham, USA). Images were acquired on an EVOS 5000 LED based fluorescence microscope.

### 2.7. Western Blotting

Cells were lysed in Laemmli buffer, and samples were subjected to standard SDS-PAGE. Separated proteins were transferred to nitrocellulose membranes (Hybond ECL; GE Healthcare, Marlborough, USA). Milk (5%) diluted in Tris-buffered saline containing 0.5% Tween 20 was used for blocking.

### 2.8. Biarsenic Labelling and Live Imaging of Infected Cells

The day before the experiments, 5 × 105 MDCK cells were plated on the ibidi µ-Slide 4 Well Ph+ Poly-L-Lysine (Cat# 80444, Sigma Aldrich; St. Louis, USA) and grown using DMEM 10 % FBS. The day after, MDCK cells were infected with HA-TC IAV at 5 MOI. Three h post infection, after washing with PBS, HA staining was performed using TC-FlAsH II In-Cell Tetracysteine Tag Detection Kit (Green Fluorescence), for live-cell imaging (Thermofisher; Cat# T34561; Waltham, USA), according to the manufacture’s protocol. Briefly, 2.5 µM biarsenic dye was incubated with infected cells for 30 min. This was followed by 3 washes with BAL buffer and 1 wash with PBS (10 min each). After the staining, the cells were kept in live cells imaging media which contained 0.25 µM biarsenic dye (Thermo Fisher; Cat# A14291DJ; Waltham, USA) for all the duration of the experiment. Live imaging was performed using ZEISS SLM 880 Airyscan confocal microscope at the Microscopy Shared Research Facility in the Icahn School of Medicine at Mount Sinai. The confocal analysis was conducted using a Plan Apochromatic × 63 /1.4 oil objective lens. A total of 45 Z stacks of 0.45 µm each were recorded at 10-min interval starting from 4 h up to 16 h post infection.

### 2.9. Imaging Data Analysis

Imaris software (Oxford instruments; Concord, USA) was used to analyze live imaging data series. “Surpass” option was selected to open image series folder and kept in “3D” view. “Surface” was assigned to HA signal and “Threshold” was adjusted to remove background signal, and False color was assigned to surface (Figure 3). Video files were generated by stitching the Z stack composite images of the time series, and snapshots were taken at indicated time points.

### 2.10. Software and Statistical Analysis

Molecular graphics of the crystal structure of the 1934 Human H1 Hemagglutinin (pdb id: 1RU7) were prepared by using the PyMOL molecular graphics system (version1.8; Schrödinger, LLC). All data were analyzed with GraphPad Prism software (GraphPad Software, Inc, San Diego, USA). An unpaired Student’s t-test was used to determine significant differences in viral titers, FACS and immunofluorescence analysis. Values were considered statistically significant when * *p* < 0.05, ** *p* < 0.01 and *** *p* < 0.001, ns > 0.05 (not significant). Data are given as mean ±SD; “n” refers to the sample size.

## 3. Results

### 3.1. Strategy for Generation of Reporter IAV Expressing Hemagglutinin with Tetra-Cysteine Tag

**Figure 1:**
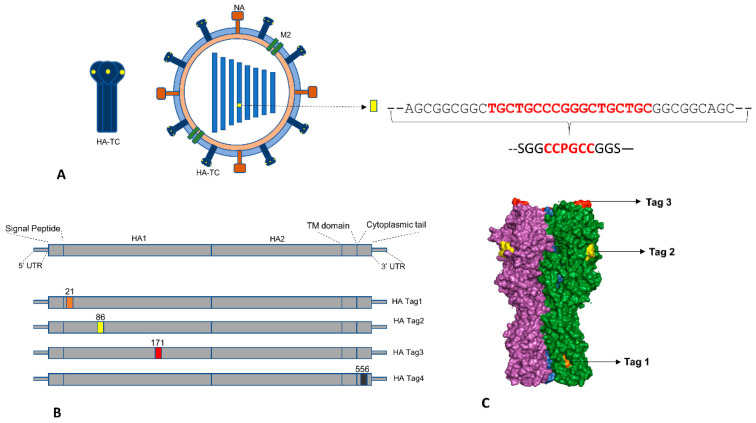
Scheme for generation of reporter influenza A virus (IAV) with Tetra-Cysteine tag inserted in hemagglutinin (HA): (**A**) Shows HA-TC reporter virus outline and nucleotide sequence inserted into genomic segment 4 which encodes for HA. The Red font indicates the Tetracysteine Tag and Black font indicates the linker sequences. (**B**) Shows sequence features of IAV HA and amino acid positions where TC tag was inserted in different HA-TC constructs. (**C**) Shows PyMOL generated 3D render of HA trimer (Magenta, Green and Blue monomers) showing sites for TC tag insertion (Tag 1: Orange; Tag 2: yellow; Tag 3: Red) in HA-Tag2 construct.

Influenza HA protein is a membrane protein expressed in a trimeric form on the host cell surface and IAV virion envelope. In three-dimensional form, it has a highly variable globular head domain and relatively conserved stalk region [2]. Previous studies have shown that HA is permissible for insertion of foreign genetic elements at specific sites and rescue of replication competent recombinant influenza strains [4,5,6]. We chose sites for TC tag insertion based on selected published reports (Table S2). Overall, we used the HA gene of Influenza A virus A/Puerto Rico/8/1934(H1N1) (GenBank: AFM71846.1) to generate four constructs with TC tag insertion at amino acid (aa) position 21, 86, 171, and 556, respectively, and denoted them HA-Tag1 to HA-Tag4 (Figure 1B). The functional domains of HA corresponding to these sites are shown in Figure 1A and Table S1. The TC tag sequence has rigid, bulky amino acids such as Proline and Cysteine; hence, to provide flexibility at the insertion site, we added SGG and GGS aa linkers at 5’ and 3’ end of the Tag (Figure 1A). For inserting the nucleotide sequence coding for the TC tag with linkers into HA sequence, we adapted PCR based splicing method described by Horton et al. [21] (Figure S1). Prior to attempting IAV rescue with HA-TC constructs, we tested the ability of these constructs to form virus-like particles (VLPs). For this, we used the method described earlier where expression plasmids of HA, NA, and M segments are co-transfected into mammalian cells leading to secretion of VLPs into culture supernatant [22]. We measured VLP formation by western blotting for secreted HA in the cell culture supernatant and cell lysates and tested the ability of HA-TC VLPs in cell culture supernatant to agglutinate sheep RBCs. We observed that compared to wild type HA, the Tag4 construct showed comparable HA titer and the ability to express in culture supernatant, whereas Tag2 showed reduced HA titer (Figure S2A,B). Tag1 and Tag3, although showed reduced expression in the cell culture supernatant, were unable to cross-link RBCs in HA titer assay.

### 3.2. Rescue and Characterization of Reporter PR8 IAV Expressing HA-TC Protein

**Figure 2:**
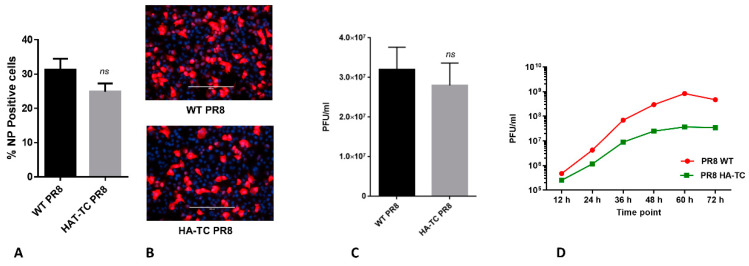
Rescue and characterization of reporter IAV expressing HA-TC protein: (**A**,**B**) Madin–Darby canine kidney (MDCK) cells were infected with WT or HA-TC PR8 IAV at MOI of 5 and 3 h post infection immunostaining was performed using anti-NP specific primary and alexa-568 (red) secondary. Nuclei were co-stained with DAPI (Blue). NP positive cells were counted from 5 independent fields and ratio over DAPI positive cells was calculated. Percentage NP positive cells were plotted on the graph. (**C**) MDCK cells were infected with WT and HA-TC PR8 IAV at MOI of 1. After 16 h post infection, culture supernatant was harvested, and virus titer was measured by plaque assay. The titers were plotted on the graph. (**D**) In a time course experiment, MDCK cells were infected with WT or HA-TC PR8 IAV at MOI of 0.02. Culture supernatants were collected at 12h intervals up to 72 h. Virus titer was measured by plaque assay, and mean titers were plotted on the graph. Data show mean ±S.D. from representative experiment (*n* = 3) of at least three independent experiments.

Based on the negative results on Tag1 and Tag 3 HA constructs to form VLPs, we chose Tag 2 and Tag 4 HA constructs to attempt recombinant IAV rescue. For this, we used 8 plasmid system as described earlier [20]. Surprisingly, Tag 4 HA construct, although showing VLP formation ability comparable to wild type (WT) HA, failed to result in recombinant IAV rescue. On the other hand, Tag2 HA reporter construct resulted in rescue of recombinant IAV. Insertion of TC tag into genome segment 4 (which expresses HA) was confirmed by sequencing of viral genomic RNA. We modeled insertion site of TC tag in HA Tag2 construct by PyMOL software, which showed that it will be exposed on the HA trimer surface and easily accessible to biarsenical dyes (Figure 1C). Next, we compared the replication competence of Tag2 HA expressing virus (called HA-TC PR8 from here on), in comparison to WT PR8 IAV. For this, we first tested HA-TC PR8 virus ability to enter MDCK cells in immunofluorescence-based assay (Figure 2A,B). Subsequently, we tested ability of HA-TC PR8 virus to express HA at the cell surface at late stages of viral replication cycle (Figure 2C). In both assays, HA-TC PR8 virus was comparable to WT PR8 IAV, with marginally attenuated (statistically not significant) phenotype. Next, we compared HA-TC PR8 virus with WT PR8 IAV in a multicycle replication experiment. Here, HA-TC PR8 virus showed an order of magnitude reduced virus titer on day 3 of infection (Figure 2D). This may have resulted due to amplification of marginal differences in entry of HA-TC PR8 and WT PR8 IAV, over multiple replication cycles. Influenza A viruses expressing different reporter genes (including tetra cysteine tag in NS1) display marginally attenuated phenotype in vitro as well as in vivo [12,20,23]. Thus, in the light of previous studies, slight attenuation of HA-TC IAV is not unexpected. Overall, results suggested that HA-TC PR8 is replication competent and suitable for single replication cycle experiments; however, in multicycle experiments, it shows attenuated phenotype.

### 3.3. Four Dimensional Live Imaging of HA in Cells Infected with HA-TC Reporter IAV

**Figure 3:**
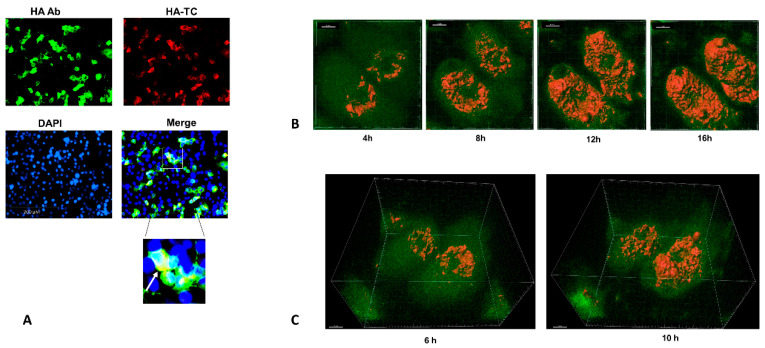
Live 4D imaging of HA in cells infected with HA-TC reporter IAV: (**A**) MDCK cells were infected with HA-TC PR8 at MOI of 1. On 12 h post infection, cells were subjected to immunostaining with anti-HA primary and alexa-488 (green) secondary. Subsequently cells were stained with ReAsh dye as per the manufacturer’s instruction. Finally, fixed cells were observed under fluorescence microscope at 40× objective. The inset shows colocalization between green (anti-HA antibody) and red (ReAsh dye) staining, marked by white arrow. (**B**) MDCK cells were infected with HA-TC PR8 at MOI of 5. On 3 h post infection, HA staining was performed using FIAsh dye as described in Methods. Live imaging was performed using confocal microscope. Z stacks were recorded at 10-min interval starting from 3 h up to 16 h post infection. Imaging data was analyzed and converted to a video using Imaris software. The Figure shows snapshots at indicated time points from the live imaging video (Video S1). HA signal above background was rendered in 3 dimensions given orange color using Imaris software. (**C**) Shows 3D perspective of HA staining from the top of the cells at indicated time points post infection. This was generated by integrating Z stack of confocal images and processing it into a 3D movie using Imaris software (Video S2).

After characterizing replication competence of HA-TC PR8, we tested expression of TC tag in infected cells by biArsenic dye labelling and fluorescence microscopy. For BiArsenic dye labelling, we used reagents from Invitrogen (#T34561/2), which include red fluorescent ReAsh and green fluorescent FIAsh dyes for this application. We performed immunofluorescence assay in HA-TC PR8 infected MDCK cells using anti-HA monoclonal Ab. Subsequently, we performed biarsenic labelling of HA-TC PR8 using ReAsh dye. The results indicated complete colocalization of green and red signals obtained by anti-HA Ab and ReAsh dye, confirming HA specific staining by ReAsh dye (Figure 3A). This experiment was performed with FIAsh dyes also and similar result was obtained. Next, we tried to perform live imaging of FIAsh biarsenic dye labelled HA-TC in reporter IAV infected cells. For this, we infected MDCK cells at MOI of 5- and 3-h post infection we introduced FIAsh dye into infected cells. Excess dye was washed off, and a limited amount was maintained during infection to stain newly synthesized HA protein. We performed 4-dimensional live imaging of FIAsh signal from these cells using confocal microscopy from 4 to 16-h post infection period (Video S1, S2, S3). Results showed HA signal from perinuclear region at 4 h post infection. As the infection proceeded, HA signal increased, indicating accumulation of newly synthesized protein (Figure 3B). HA translocation toward periphery of the cell as well as apical surface was also observed during late time points (12 h, 16 h) (Figure 3C). Results from this experiment showed that HA-TC PR8 is a useful tool for visualizing HA translocation in infected cells by live imaging. Using this tool, it is possible to appreciate accumulation of HA protein as well as its lateral and apical transport as the infection proceeds.

### 3.4. Effect of ER-Golgi Transport Inhibitors on HA Trafficking in IAV Infected Cells

**Figure 4:**
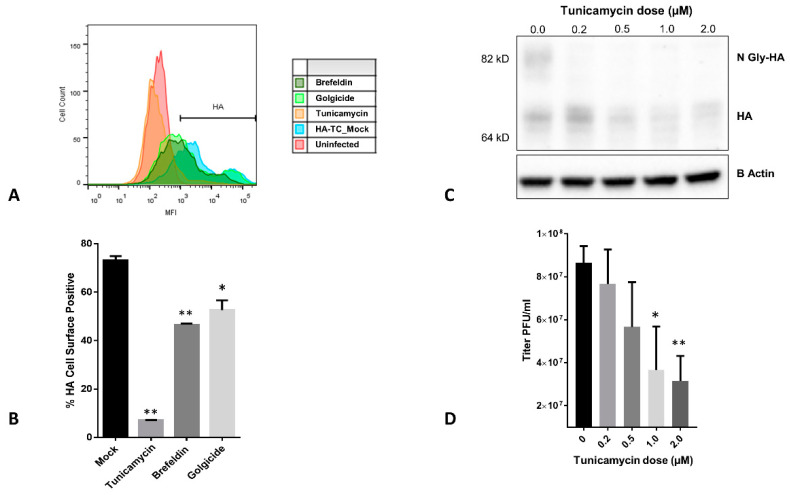
Inhibition of intracellular transport of HA by chemical inhibitors of ER-Golgi transport: (**A**) MDCK cells were infected with WT PR8 IAV at MOI of 2 and 3 h post infection, indicated drugs were introduced into the medium. On 9 h post infection, cells were stained with anti-HA primary and alexa-488 secondary antibody and subjected to flow cytometry. Data was analyzed using FlowJo software and Histogram shows HA signal under different conditions. (**B**) Percentage of HA positive cells from the FACS experiment were calculated and plotted on the graph. (**C**) MDCK cells were infected with WT PR8 IAV at MOI of 2 and 3 h post infection indicated drugs were introduced into the medium. On 9 h post infection, cells were harvested in SDS-PAGE buffer and subjected to western blotting using anti-HA and anti-Actin antibodies. (**D**) MDCK cells were infected with WT PR8 IAV at MOI of 0.02, and 3 h post infection, tunicamycin was introduced into the medium at indicated doses. Culture supernatant was harvested 48 h later, and viral titer was measured by plaque assay, as shown in the graph. Values were considered statistically significant when * *p* < 0.05, ** *p* < 0.01 and *** *p* < 0.001, ns > 0.05 (not significant). Data are given as mean ±SD; “n” refers to the sample size.

Influenza HA is a transmembrane protein, which is translated in the ER where it undergoes post translational modifications and travels through ER-Golgi network to the cell membrane [1]. We tested inhibitors of these events, and their effect on HA translocation to the cell surface. Tunicamycin is an inhibitor of N-linked protein glycosylation in the ER, and at higher concentrations, it leads to ER stress [24]. Brefeldin A inhibits ER to Golgi transport of the proteins [16]. Golgicide is an inhibitor of Arf1 GTPase, which is required for intra-Golgi transport of proteins [17]. Thus, in sequence of events in HA translation to transport, Tunicamycin will act first, followed by Brefeldin and Golgicide and should affect HA transport in that order. To test this, we exposed infected MDCK cells with these drugs and measured HA expression on cell surface by Flow cytometry. As expected, all three had inhibitory effect; however, Tunicamycin was most effective in reducing HA expression on surface of infected cells (Figure 4A,B). To validate the mechanism by which Tunicamycin affected HA transport, we performed western blotting to see glycosylation status of HA in presence of increasing dose of the drug. We observed that glycosylated form of HA was absent at as low as 0.2 µM dose of Tunicamycin (Figure 4C). Effect of tunicamycin treatment also reflected on multicycle replication of the IAV, which decreased upon increasing dose of the drug (Figure 4D). The 2 µM dose of tunicamycin showed maximum effect on HA transport to cells surface, inhibition of HA glycosylation and virus replication; hence, we chose this drug and concentration for further experiments.

### 3.5. Quantiative Live Imaging of HA Transport Retardation by Tunicamycin

**Figure 5:**
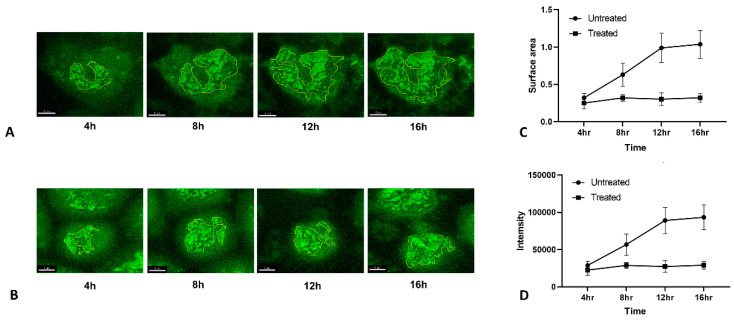
Live imaging of HA transport retardation by Tunicamycin (**A**,**B**) MDCK cells were infected with HA-TC PR8 IAV at MOI of 5. After 3 h post infection, HA staining was performed using FIAsh dye as described in methods. Live imaging was performed using confocal microscope. Z stacks were recorded at 10-min interval starting from 3 h up to 16 h post infection. Imaging data was analyzed and converted to a video using Imaris software. A shows imaging done in control condition, where B shows imaging done in presence of Tunicamycin at 2 µM concentration. (**C**,**D**) The surface area and intensity of HA signal under control and drug treated condition at different time points was measured by using Imaris software. Values were recorded from 3 independent fields and mean ±SD were plotted on the graphs shown in the figure.

After testing HA-TC PR8 IAV’s utility in 4-dimensional tracking of HA in infected cells, we explored possibility of using this tool for quantifying changes in HA accumulation and translocation during infection under different conditions. For this, we performed live imaging in HA-TC PR8 IAV infected MDCK cells in presence or absence of Tunicamycin (Figure 5A,B). Using image analysis tools, we measured two parameters using image analysis tools: (i) Surface Area, which represents the distance traveled in 3 dimensions by HA, thus is a measure of translocation. (ii) Signal Intensity, which represents the total amount of HA present at different time points, thus is a measure of HA accumulation during infection (Figure 5C,D). We observed that in control infected cells, HA continues to accumulate up to 12 h post infection and between 12 to 16 h both HA trafficking and accumulation reaches saturation (Figure 5A). In contrast, in Tunicamycin treated cells, both HA accumulation and translocation showed marginal increase up to 8 h but afterward arrested at all time points (Figure 5B). This may result from ER stress induced by Tunicamycin treatment, which leads to overall arrest of protein translation.

## 4. Discussion

Influenza A virus replication cycle can be roughly divided into viral entry, viral protein synthesis and genome replication, viral assembly and budding [1]. The events involved in viral entry, specially role of HA in the same have been studied in great details. This includes structural studies on HA-receptor interaction and characterizing conformational changes in HA molecule during receptor mediated endocytosis [2]. Comparatively, the events from HA translation and trafficking to the viral budding sites are less understood. Live imaging of HA in infected cells can give detailed insight into these late events of viral replication. Several researchers have engineered Influenza A viruses through reverse genetics to study different aspects of viral replication. These include insertion of Fluorescent or Bioluminescent reporters in to IAV structural proteins or expression of tetra cysteine or Sortase tags that allow chemical labelling of the viral protein for fluorescence-based detection [12,14,20,23,25,26,27,28]. Here, we focused on developing a reporter virus to achieve live imaging of HA dynamics in infected cells. For this, we inserted Tetracysteine tag in the HA molecule at 4 different sites. The site 1 is close to the signal peptide and has been reported to tolerate insertion of up to 140 aa foreign peptides from *Bacillus anthracis* [6]. Site 2 is in the antigenic site B, where insertion of an 8 aa epitope from *Plasmodium yoelli* has been reported [5]. Site 3 is in the antigenic site A, where insertion of Beta amyloid antigen (7–10 aa) has been reported [29]. The site 4 was present in the cytoplasmic tail of HA and was chosen empirically. In the VLP formation assay, Tag2 and Tag4 HA constructs performed better than others, indicating insertion of foreign sequences in the cytoplasmic tail does not interfere with HA secretion and VLP formation. However, between Tag1, 2, and 3, only Tag2 formed VLPs with an efficiency comparable to wild type HA. This could be due to structural interference of TC tag with HA folding at site 1 and 2. Surprisingly, in virus rescue experiment, we were able to rescue only Tag2. The Tag4 HA, although performing well in VLP assay, did not yield replicative virus. This could be due to interference caused in the packaging sequence of HA vRNA, caused due to TC tag sequence insertion.

The rescued Tag2 HA-TC reporter IAV showed replication competence as compared to WT PR8 IAV, especially in single cycle assays. However, its titer was attenuated by 1 log in multicycle infection. This could have resulted due to incremental effect of slight attenuation in viral entry over the course of multiple replication cycles. Post translational modifications (PTM) are important for HA antigenicity; however, their role in intracellular transport and viral replication is not clear [1]. Here, we observed that HA PTM can be inhibited by Tunicamycin, at a dose as low as 0.2 µM; however, effect of Tunicamycin on multicycle viral replication was significant only at higher doses (2 µM). This effect could be due to induction of endoplasmic reticulum stress by Tunicamycin [24]. Overall, this suggested that HA PTM may not be critical for HA trafficking and viral replication. For live imaging experiments, we chose 2 µM dose of Tunicamycin, which was expected to interfere with HA trafficking due to ER stress induction. This assumption is supported by live imaging data, which showed reduced HA accumulation and translocation in presence of Tunicamycin. It has been reported that in polarized cells, HA transport occurs towards apical membrane [28]. In the live imaging experiment, we were able to observe lateral accumulation of HA at early time points, followed by apical transport at the late stages of infection. The apical transport was diminished in Tunicamycin treated cells. These experiments showed utility of HA-TC IAV in true 4-dimensional tracking of HA during IAV infection in cells.

The Tetracysteine tag has several advantages over other fluorescent protein strategies. Small size of the TC-Tag (6 amino acids, 585 Da) is less likely to interfere with the structure or biological activity of the protein of interest [7]. The HA-TC virus reported here has the TC tag inserted in the antigenic site B, and three-dimensional protein modelling suggests that the Tag is on the surface and easily accessible for conjugation with biarsenic dyes. The FlAsH-EDT2 and ReAsH-EDT2 labeling reagents are membrane-permeable and readily cross the cell membrane, allowing labeling and detection of recombinant proteins in live mammalian cells. ReAsH-EDT2 reagent is sensitive to photo-bleaching when exposed to continuous illumination. Moreover, it is more cytotoxic compared to FlAsH-EDT2. We showed specific and strong labelling of HA-TC with both dyes; however, in live imaging experiments, we preferably used FIAsh-EDT2 reagent to label the recombinant HA protein. The TC tag sequence (CCPGCC) is absent in mammalian genome, thus providing specificity. The TC tag or the biarsenic dyes are not fluorescent by themselves, and only when they conjugate together, the fluorophore is formed [7]. There are certain limitations of this tool for long term live imaging. The cells need to be infected at high MOI (equal or higher than 5) to visualize any HA signal at early time points (~3 h P.I.) IAVs have natural cytotoxicity, and at high MOI, they induce extensive cell death. Towards the end of an infection cycle, a large proportion of cells undergo apoptosis. The phototoxicity caused by intermittent exposure to confocal laser and cytotoxicity caused by IAV and biarsenic dyes make it difficult to image cells for long periods of time. Nevertheless, with optimized conditions, we were able to record events associated with HA accumulation and transport to the cell’s membrane, representing a complete replication cycle.

Looking forward there are several applications of this reporter virus that can be explored. The biarsenic dyes are available in ReAsh and FIAsh format, which can be used in sequential staining in pulse label experiment to monitor HA protein turnover. Beyond fluorescence imaging, biarsenic staining of HA can also allow FRET based cellular assays to study protein interaction with other viral or cellular factors. The biarsenic dyes are also compatible with electron microscopy, which can allow detailed imaging of HA during viral morphogenesis. As opposed to time consuming conventional immunofluorescence, HA-TC PR8 IAV allows rapid staining of HA with biarsenic dyes. This can be scaled up for high throughput screening assays to identify antiviral drugs that interfere with HA trafficking or synthesis and study host factor requirement for these processes. We have observed that the virus particles produced from HA-TC PR8 IAV infected cells in presence of biarsenic dye are fluorescently labelled and infectious, which can be used for studying virus entry events in detail. So far, lipophilic dyes have been used to study IAV entry events, which allow tracking of single virus particles and events up to the fusion of viral and cellular endosomal membrane [30]. Using a biarsenic dye labeled HA-TC PR8 IAV will allow tracking of HA even after the membrane fusion event has taken place. This tool can allow visualization of virion assembly events when used in combination with imaging of viral RNPs and other structural proteins. Overall, the HA-TC reporter IAV allows spatial and temporal tracking of HA trafficking and accumulation in IAV infected cells and can be a versatile tool in studying IAV biology and antiviral discovery.

## Supplementary Materials

The following are available online at https://www.mdpi.com/1999-4915/12/6/687/s1. Video S1: Top view of HA in HA-TC IAV infected MDCK cells from 4 to 16 h post infection. Video S2: Side view of HA in HA-TC IAV infected MDCK cells from 4 to 16 hrs post infection. Video S3: Four-Dimensional tracking of HA in HA-TC IAV infected MDCK cells from 4 to 16 hrs post infection. Figure S1: Overlap extension PCR Strategy for TC Tag insertion in HA. Figure S2: Characterization VLP formation by HA-TC constructs.
