# Supplementary figures and images for: Live Visualization of Hemagglutinin Dynamics during Infection by Using a Novel Reporter Influenza A Virus
Source: Viruses. 2020 Jun 26;12(6):687. doi: 10.3390/v12060687 (PMC7354568; doi:10.3390/v12060687)

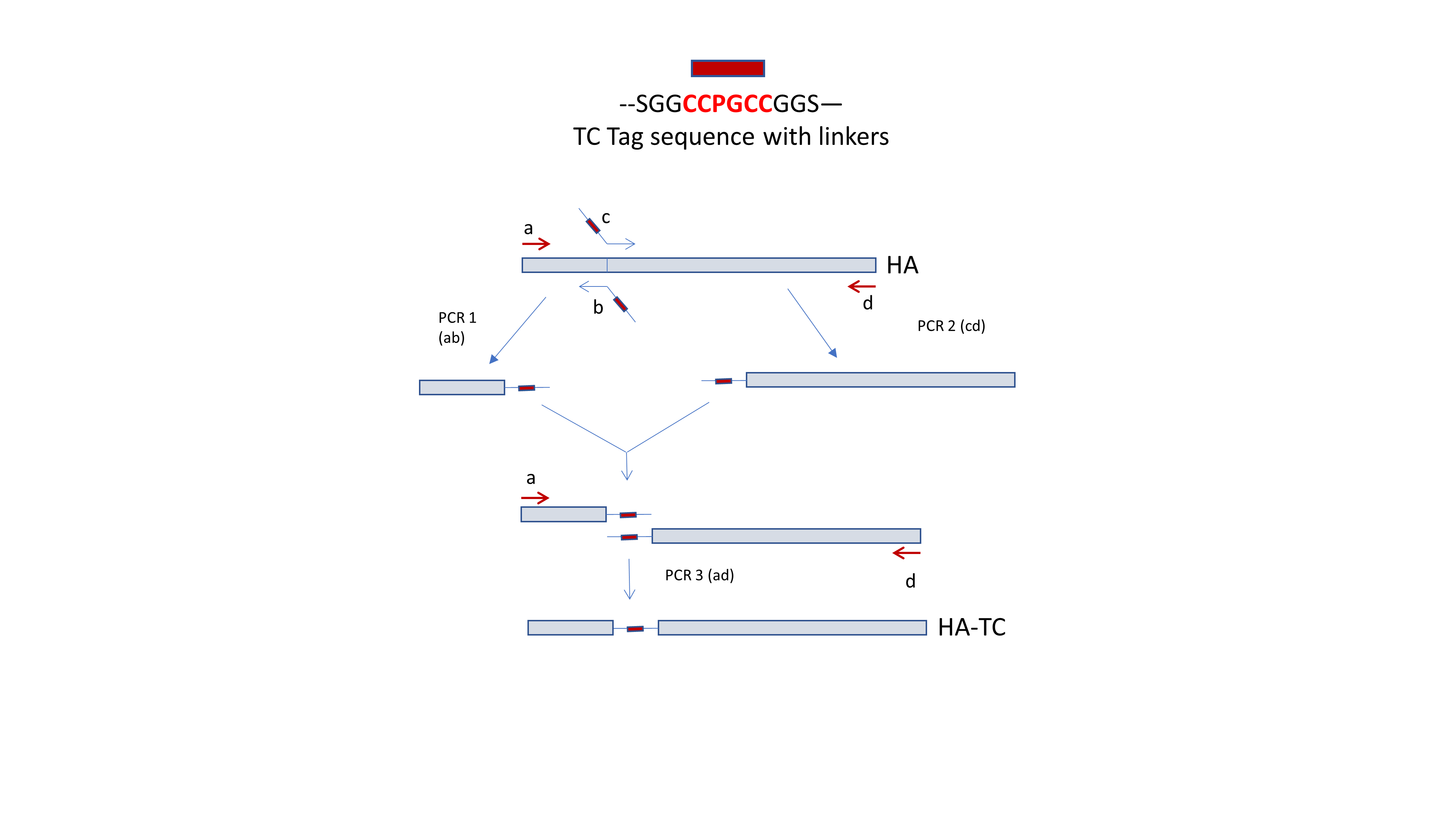

Supplement: Supplementary file 1 [file viruses-12-00687-s001.zip › viruses-844500-suppl/Supplementary Figure 1.TIF]

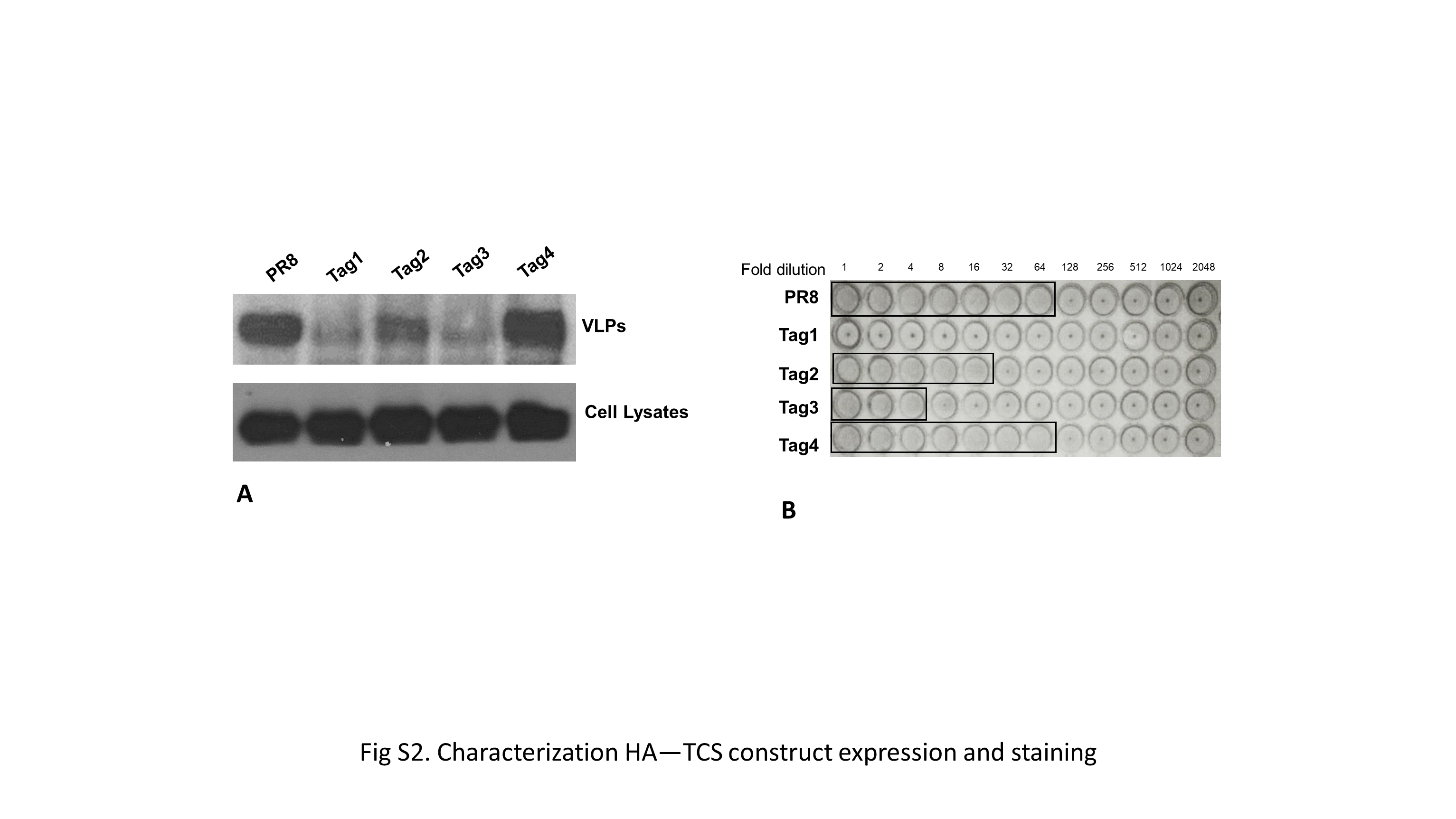

Supplement: Supplementary file 1 [file viruses-12-00687-s001.zip › viruses-844500-suppl/Supplementary Figure 2.tif]
